# Nutritional and functional values of lysed *Corynebacterium glutamicum* cell mass for intestinal health and growth of nursery pigs

**DOI:** 10.1093/jas/skab331

**Published:** 2021-11-19

**Authors:** Yi-Chi Cheng, Marcos Elias Duarte, Sung Woo Kim

**Affiliations:** Department of Animal Science, North Carolina State University, Raleigh, NC 27695, USA

**Keywords:** *Corynebacterium glutamicum*, growth performance, intestinal health, mucosa-associated microbiota, pigs, protein supplement

## Abstract

The objective was to determine the nutritional and functional values of lysed *Corynebacterium glutamicum* cell mass (CGCM) as a protein supplement and a source of cell wall fragments supporting the growth and intestinal health of nursery pigs. Thirty-two pigs (21 d of age) were allotted to four treatments (*n* = 8) based on the randomized block design with sex and initial body weight (BW) as blocks. The main effect was the dietary supplementation of lysed CGCM (0, 0.7, 1.4, and 2.1%) replacing blood plasma and fed in two phases (10 and 11 d, respectively). Feed intake and BW were measured at the end of each phase. Pigs were euthanized on day 21 to collect jejunal tissue and mucosa to evaluate intestinal health. Ileal digesta were collected to measure the apparent ileal digestibility of nutrients in diets. Data were analyzed using Proc Mixed and Reg of SAS. Increasing daily intake of CGCM increased (linear; *P* < 0.05) ADG of pigs. Increasing CGCM supplementation affected (quadratic; *P* < 0.05) the relative abundance of *Lactobacillaceae* (minimum: 26.4% at 1.2% CGCM), *Helicobacteraceae* (maximum: 29.3% at 1.2% CGCM), and *Campylobacteraceae* (maximum: 9.0% at 1.0% CGCM). Increasing CGCM supplementation affected (quadratic; *P* < 0.05) the concentrations of immunoglobulin G (maximum: 4.94 µg/mg of protein at 1.0% CGCM) and protein carbonyl (PC; maximum: 6.12 nmol/mg of protein at 1.1% CGCM), whereas linearly decreased (*P* < 0.05) malondialdehyde (MDA) in the proximal jejunal mucosa. Increasing CGCM supplemention affected (quadratic; *P* < 0.05) intestinal enterocyte proliferation rate (maximum: 13.3% at 1.0% CGCM), whereas it did not affect intestinal morphology and the nutrient digestibility. In conclusion, supplementing 1.0% to 1.2%, reducing blood plasma supplementation by 0.7% to 0.9%, respectively, increased potential pathogenic microbiota associated in the jejunal mucosa resulting in increased immune response, enterocyte proliferation, and PC concentration. However, supplementing diets with 2.1% CGCM, replacing 1.5% blood plasma, improved growth performance, and reduced MDA without affecting nutrient digestibility, intestinal morphology, and microbiota in the jejunal mucosa. In this study, based on the polynomial contrast, supplementing 1.0% to 1.2% CGCM suppressed the benefits from blood plasma, whereas supplementing 2.1% CGCM showed functional benefits of CGCM with similar effects from blood plasma supplementation.

## Introduction

Soybean meal has been predominantly used as a protein supplement in pig production. However, the antinutritional factors such as allergenic proteins, trypsin inhibitors, and flatulence producing oligosaccharides limit its use in nursery pig diets (Kim et al., 2003; Hong et al., 2004; Taliercio and Kim, 2013). Previous studies have shown that animal protein supplements, including blood plasma and fish meal, enhance growth performance, nutrient digestibility, and intestinal health of nursery pigs (Kim and Easter, 2001; Bosi et al., 2004; Weaver et al., 2014). Blood plasma contains 17% to 23% immunoglobulin (Ig) G, which is sufficient to enhance the immune system by inhibiting the adherence of antigens in the intestinal mucosa and, consequently, improving the growth performance (Touchette et al., 2002; Pierce et al., 2005; Tran et al., 2014). However, animal protein supplements are expensive and short in supply in animal feeding (USDA, 2019). The rising cost of pig diets leads nutritionists to increase attention to alternatives replacing conventional animal protein supplements (Øverland et al., 2001; Carlson et al., 2005; Kim et al., 2010).

Alternative protein supplements with adequate nutritional values and bioavailability include single cell protein (SCP), processed vegetable protein, and insect protein (Kim et al., 2019). The SCP, including yeast, microalgae, and bacterial cells, contains high levels amino acids (AA), fats, and vitamins (Kihlberg, 1972; Michalak et al., 2015; Lopes et al., 2017). However, intact SCP supplemented in animal diets may cause reduced growth performance, intestinal health, and nutrient digestibility due to indigestible cell wall (Rumsey et al., 1991; Zhang et al., 2013; Cruz et al., 2020) and the potential endogenous toxins (Farstad, 1977) reducing nutrient digestibility and causing intestinal challenges to young pigs (Kim et al., 2019). The utilization of chemical, enzymatic, and physical processes to lyse bacterial cell wall can release digestible and functional contents from the cell (Namioka et al., 1991; Ugalde and Castrillo, 2002; Becker and Richmond, 2004). In addition, the use of non-harmful bacteria such as *Methylococcus capsulatus* (Ritala et al., 2017) and *Corynebacterium glutamicum* (Lee et al., 2016) can overcome the potential toxicity of SCP.

*Corynebacterium glutamicum* is a Gram-positive bacteria, commonly used to produce AA, and generally recognized as safe (AAFCO, 2008). After the production of AA, *Corynebacterium glutamicum* cell mass (CGCM) is removed by filtration and then disposed of or used as fertilizers (Kircher and Pfefferle, 2001). However, CGCM contains similar concentrations of protein and AA to animal protein supplements (Zhang et al., 2013) and could partially replace some conventional animal protein supplements, such as fish meal, poultry meal, and blood plasma in feeding pigs. Selected components of bacterial cell called cell-wall glycopolymers (CWGs) including peptidoglycan (PGN), teichoic acid (TA), and lipoprotein can show immunomodulatory functions in the intestine of pigs (Namba et al., 1981; Namioka et al., 1991; Akira et al., 2006) by activating immune cells (Katayama et al., 2011; Poulsen et al., 2018). Additionally, the surface layer proteins (Slp) and TA play important roles in pathogen exclusions (Johnson-henry et al., 2007; Oelschlaeger, 2010). In order to enhance the possible use of such cell wall components, fragmentation or lysis of cell walls can enhance their roles in immune modulation (Humann and Lenz, 2009; Shen et al., 2009), in addition to the release of cell contents as a source of nutrients for pigs (Øverland et al., 2010).

It is hypothesized that lysed CGCM as a protein supplement, replacing blood plasma, would provide nutritional and functional benefits by increasing nutrient utilization and by enhancing intestinal health in nursery pigs. To test the hypothesis, the objectives were to evaluate nutritional values of lysed CGCM as a novel protein supplement and to evaluate the functional roles of lysed CGCM to enhance the intestinal health of nursery pigs.

## Materials and Methods

The experimental protocol was approved by the Institutional Animal Care and Use Committee of North Carolina State University.

### Preparation of lysed CGCM

Lysine broth including intact CGCM was obtained from CJ Bio (Fort Dodge, IA). The lysine broth containing 15% to 20% dry matter (DM) was centrifuged at 3,100 × *g* for 15 min to obtain pellets. Deionized water was added and vortexed for 30 s or longer until pellets were reconstituted in deionized water. Eighty liters of CGCM in deionized water went through the lysis using a French press (120 L/h capacity, NS3006H, Niro Soavi S.p.A., Parma, Italia) at 900 bars for 4 s at 30 °C with a maximum allowance of 70 °C in the holding tube. After the holding time, the CGCM in deionized water flowed to a serpentine tube immersed into chilled water for rapid cooling. The lysis process was completed in four cycles in a continuous system (Biomanufacturing Training and Education Center, North Carolina State University). The lysed CGCM was dried using a freeze dryer (24DX48, Virtis, Gardiner, NY). The lysing process was based on the study by Rumsey et al. (1991).

**Figure 1.**
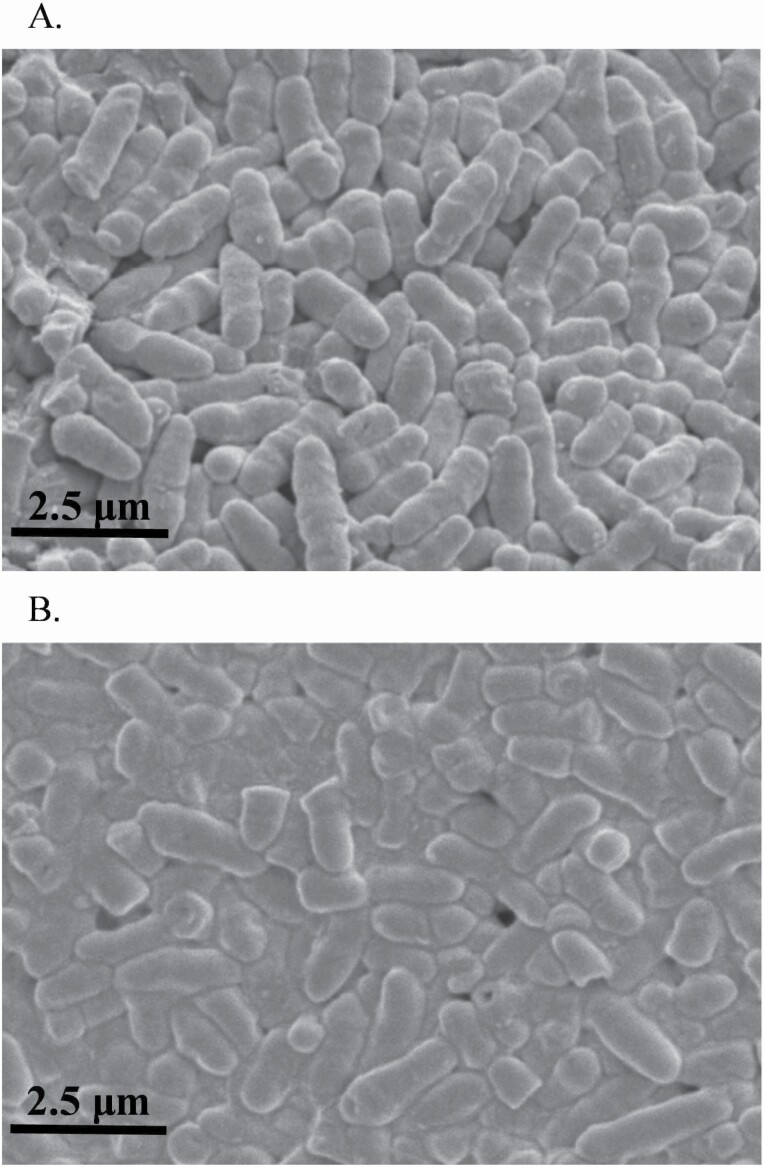
Images of *Corynebacterium glutamicum* cell mass before (A) and after (B) homogenization taken by Scanning Electron Microscope (S3200N, Hitachi, Japan).

Images of CGCM before and after the lysis were taken using a variable pressure scanning electron microscope (S3200N, Hitachi, Japan) at the 5 kV efficient voltage and 10 mm working distance to obtain the percentage of lysed cells. Each image of CGCM from before and after the lysis (Figure 1) was evenly divided into eight sections. The intact and lysed cells were counted to calculate the percentage of lysed cells in each section (Table 1). Protein and AA composition of intact and lysed CGCM (Table 1) were measured in triplication at Agricultural Experimental Station Chemical Laboratories, University of Missouri (Columbia, MO).

**Table 1.** Percentage of lysed cells and analyzed nutrient composition in *Corynebacterium glutamicum* cell mass (CGCM) (as-is basis)

	CGCM		
	Intact	Lysed	SEM
Lysed CGCM, %^1^	4.3	58.8	2.0
Crude protein, %	79.9	80.5	0.3
Indispensable amino acids, %			
Arginine	4.5	4.5	0.0
Histidine	1.7	1.7	0.0
Isoleucine	3.8	3.8	0.0
Leucine	5.9	6.0	0.1
Lysine	7.4	4.6	1.4
Methionine	1.4	1.4	0.0
Phenylalanine	3.1	3.1	0.0
Threonine	3.5	3.6	0.0
Tryptophan	0.3	0.8	0.2
Valine	5.2	5.3	0.0

^1^Each image from intact and lysed CGCM was evenly divided into eight sections and the numbers of intact and lysed cells were counted to calculate the percentage of lysed cells in each section.

### Animals, experimental design, and diets

Thirty-two nursery pigs (16 barrows and 16 gilts) at 21 d of age with initial body weight (BW) of 7.2 ± 0.6 kg were used for the experiment. Pigs were allotted to four dietary treatments (*n* = 8) based on the randomized complete block design with initial BW (light and heavy) and sex (gilts and barrows) as blocking criteria. All pigs were housed in individual pens and had ad libitum access to water and the assigned experimental diets for 21 d based on two phases: phase 1 (days 0 to 10) and phase 2 (days 11 to 21). Dietary treatments consisted of a basal diet with lysed CGCM at four levels (0, 0.7, 1.4, and 2.1%) of CGCM replacing blood plasma, based on Lys content (i.e., at 6.9:4.6 blood plasma to lysed CGCM Lys ratio). Therefore, up to 1.5% blood plasma was replaced by 2.1% CGCM. All experimental diets were formulated to meet or exceed the nutrient requirements based on the study by NRC (2012). The composition of mash experimental diets is shown in Table 2. Titanium dioxide (0.4%) was added to the feed as an indigestible external marker and fed during the last 7 d of the experiment.

**Table 2.** Composition of experimental diets (as-fed basis)

Item	CGCM							
	Phase 1				Phase 2			
	0%	0.7%	1.4%	2.1%	0%	0.7%	1.4%	2.1%
Feedstuff, %								
Yellow dent corn, ground	42.8	42.5	42.2	41.9	50.3	50.0	49.7	49.4
Whey permeate	24.0	24.0	24.0	24.0	15.0	15.0	15.0	15.0
Poultry fat	2.15	2.27	2.39	2.51	1.98	2.10	2.22	2.34
Soybean meal, 48% CP	20.0	20.0	20.0	20.0	23.0	23.0	23.0	23.0
Poultry meal	3.00	3.00	3.00	3.00	2.80	2.80	2.80	2.80
Fish meal	2.00	2.00	2.00	2.00	-	-	-	-
Blood plasma	2.90	2.40	1.90	1.40	4.08	3.58	3.08	2.58
CGCM^1^	0.00	0.70	1.40	2.10	0.00	0.70	1.40	2.10
l-Lys	0.72	0.72	0.71	0.71	0.43	0.43	0.42	0.42
dl-Met	0.31	0.31	0.32	0.32	0.18	0.17	0.18	0.18
l-Thr	0.27	0.27	0.26	0.26	0.12	0.12	0.11	0.11
l-Trp	0.05	0.05	0.06	0.06	0.00	0.00	0.01	0.01
Salt	0.25	0.25	0.25	0.25	0.25	0.25	0.25	0.25
Dicalcium phosphate	0.65	0.65	0.65	0.65	0.85	0.84	0.84	0.84
Limestone	0.70	0.70	0.70	0.70	0.85	0.84	0.84	0.84
Vitamin premix^2^	0.03	0.03	0.03	0.03	0.03	0.03	0.03	0.03
Mineral premix^3^	0.15	0.15	0.15	0.15	0.15	0.15	0.15	0.15
Calculated composition								
DM, %	90.6	90.7	90.8	90.9	90.2	90.3	90.4	90.4
ME, kcal/kg	3,401	3,401	3,401	3,401	3,401	3,401	3,401	3,401
SID^4^ Lys, %	1.50	1.50	1.50	1.50	1.35	1.35	1.35	1.35
SID Met+Cys, %	0.83	0.83	0.83	0.83	0.74	0.74	0.74	0.74
SID Trp, %	0.25	0.25	0.25	0.25	0.23	0.23	0.23	0.23
SID Thr, %	0.88	0.88	0.88	0.88	0.79	0.79	0.79	0.79
Ca, %	0.85	0.85	0.85	0.85	0.80	0.80	0.80	0.80
STTD^5^ P, %	0.46	0.46	0.45	0.44	0.42	0.42	0.41	0.40
Analyzed composition, %								
DM^6^	90.4	90.2	90.2	90.4	90.4	89.7	90.1	90.0
CP	22.4	23.3	23.1	23.2	22.7	23.6	23.7	23.8
EE^7^	4.22	4.53	4.62	4.83	4.41	4.10	4.37	4.29

^1^CGCM, *Corynebacterium glutamicum* cell mass.

^2^The vitamin premix provided the following per kilogram of complete diet: 6,613.8 IU of vitamin A as vitamin A acetate, 992.0 IU of vitamin D_3_, 19.8 IU of vitamin E, 2.64 mg of vitamin K as menadione sodium bisulfate, 0.03 mg of vitamin B_12_, 4.63 mg of riboflavin, 18.52 mg of _D_-pantothenic acid as calcium pantothenate, 26.45 mg of niacin, and 0.07 mg of biotin.

^3^The trace mineral premix provides the following per kilogram of complete diet: 33.0 mg Mn as manganous oxide, 109.5 mg of Fe as ferrous sulfate, 109.5 mg of Zn as zinc sulfate, 16.5 mg of Cu as copper sulfate, 0.3 mg of I as ethylenediamine dihydroiodide, and 0.3 mg of Se as sodium selenite.

^4^SID, standardized ileal digestible.

^5^STTD, standardized total tract digestible.

^6^DM, dry matter.

^7^EE, ether extract.

### Growth performance and fecal score

The BW and feed intake of each pig were recorded at days 0, 10, and 21 to calculate BW, ADG, ADFI, G:F, and daily CGCM intake. Fecal scores were recorded individually on every odd day from days 3 to 19. Fecal scores were: (1) very hard and dry stool, (2) firm stool, (3) normal stool, (4) loose stool, and (5) watery stool with no shape following Weaver and Kim (2014) and Guo et al. (2015).

### Sample collection

After 21 d of feeding, all pigs were euthanized by exsanguination after the penetration of a captive bolt to the head to collect samples. Sections of proximal (1.5 m after the pyloric duodenal junction) and distal (1.5 m before the ileocecal junction) jejunum were collected and rinsed with 0.9% saline solution to collect mucosa and tissue. The jejunal mucosa was collected into 2 mL microcentrifuge tubes and immediately frozen in liquid nitrogen, and then stored at −80 °C for determination of immune status, oxidative stress markers, and microbiota. Proximal and distal jejunum tissues (5 cm) were collected into 50 mL polypropylene tubes with 40 mL of 10% buffered formaldehyde solution for histological evaluation. Ileal digesta was collected into 150 mL containers placed on ice and then stored at −20 °C for analysis of apparent ileal digestibility (AID).

### Relative abundance and diversity of jejunal mucosa-associated microbiota

Distal jejunal mucosa samples were used to extract DNA using QIAamp Fast DNA Stool Mini Kit (#51604, Qiagen, Germantown, MD) following the description from Duarte et al. (2020). Samples were sent to Mako Medical Laboratories (Raleigh, NC) for microbial sequencing using the 16S rRNA technique. Libraries were prepared with the Ion Xpress Plus Fragment Library Kit (cat. no. 4471269, Thermo Fisher Scientific) from the expanded target regions, and the IonCode Barcode Adapters 1-384 Kit (cat. no. A29751, Thermo Fisher Scientific) was used for barcoding and multiplexing of the prepared libraries. The libraries were quantified with the Ion Universal Library Quantitation Kit (cat. no. A26217, Thermo Fisher Scientific) and samples were diluted to equivalent concentration and pooled into multiplexed libraries for template preparation. Template preparation and chip loading were performed using the Ion Chef instructions and sequencing was performed on the Ion S5 system with the Ion 520 & Ion 530 Kit-Chef (cat. no. A30010, Thermo Fisher Scientific) and the Ion 530 Chip Kit-4 Reactions (cat. no. A27763, Thermo Fisher Scientific). Sequences were processed using the Torrent Suite Software (version 5.2.2) (Thermo Fisher Scientific). Using the Ion Reporter Software Suite (version 5.2) of bioinformatics analysis tools applied on sequence data analysis, alignment to GreenGenes and MicroSeq databases, alpha and beta diversity plot generation, and OTU table generation. Following Duarte et al. (2021), OTU data were used to calculate the relative abundance. OTU with relative abundance <1.0% were combined as Others for statistical analysis.

### Immune status and oxidative stress

Jejunal mucosa samples (1 g) from microcentrifuge tubes were taken and added with 2 mL of PBS solution into 5 mL polypropylene tubes. Mucosa samples were homogenized using a tissue homogenizer (Tissuemiser, Thermo Fisher Scientific Inc., Rockford, IL) for 30 s on ice and transferred to a new 2 mL microcentrifuge tube for centrifugation for 15 min at 14,000 × *g* at 4 °C as described by Holanda et al. (2020). The supernatant was divided into eight sets of 0.25 mL into polypropylene tubes and stored at −80 °C for further analysis.

The concentrations of total protein, tumor necrosis factor alpha (TNFα), interleukin-8 (IL-8), malondialdehyde (MDA), protein carbonyl (PC), IgA, and IgG were analyzed by the colorimetric method and the absorbance was measured on a plate reader (Synergy HT, BioTek Instruments; Winooski, VT) and the Gen5 Data Analysis Software (BioTek Instruments).

The concentration of total protein was analyzed by using Pierce BCA Protein Assay Kit (#23225, Thermo Fisher Scientific, Waltham, MA) following procedures of Jang and Kim (2019). Mucosa samples were diluted (1:60) to reach the working range of 20 to 2,000 μg/mL. The absorbance was measured at 562 nm. The concentration of total protein was calculated by the standard curve and used to normalize the concentrations of TNFα, IL-8, IgA, IgG, MDA, and PC.

The concentration of TNFα was analyzed in proximal and distal mucosa using the Porcine TNFα Immunoassay Kit (#PTA00, R&D Systems; Minneapolis, MN) as described by Chen et al. (2017). The working range of standards was 0 to 1,500 pg/mL. The absorbance was measured at 450 nm. The concentration of TNFα was calculated by the standard curve and described as pg/mg of protein. The concentration of IL-8 was analyzed using the Porcine IL-8/CXCL8 Immunoassay Kit (#P8000, R&D Systems) following Moita et al. (2021). Mucosa samples were diluted (1:8) to reach the working range of standards from 0 to 4,000 pg/mL. The absorbance was measured at 450 nm. The concentration of IL-8 was calculated by the standard curve and described as ng/mg of protein. The concentration of IgA was analyzed using the pig ELISA kit (# E101-102, Bethyl Laboratories; Montgomery, TX) following the description from Weaver and Kim (2014) in serum. Mucosa samples were diluted (1:500) to reach the working range of standards from 15.6 to 1,000 ng/mL. The absorbance was measured at 450 nm. The concentration of IgA was calculated by the standard curve and described as μg/mg of protein. The concentration of IgG was analyzed using the pig ELISA kit (# E101-104, Bethyl Laboratories) following Duarte et al. (2020). Mucosa samples were diluted (1:1,000) to reach the working range of standards from 7.8 to 500 ng/mL. The absorbance was measured at 450 nm. The concentration of IgG was calculated by the standard curve and described as μg/mg of protein. The concentration of MDA was analyzed using Thiobarbituric Acid Reactive Substance MDA Quantitation Assay Kit (#STA-330, Cell Biolabs, Inc., San Diego, CA) following Zhao and Kim (2020). The working range of MDA standards was 0 to 125 μM. The absorbance was measured at 532 nm. The concentration of MDA was calculated by standard curve and described as µmol/mg of protein. The concentration of PC was analyzed using Protein Carbonyl ELISA Kit (#STA-310, Cell Biolabs, Inc.) following the description from Duarte et al. (2019). All samples were diluted to reach the protein concentration at 10 μg/mL to meet the working range of standards from 0.375 to 7.5 nmol/mg protein. The absorbance was measured at 450 nm. The concentration of PC was calculated by the standard curve and described as nmol/mg of protein.

### Intestinal morphology and crypt cell proliferation

Two sections of proximal and distal jejunum were cut and placed into cassettes and sent to North Carolina State University College of Veterinary Medicine Histopathology Lab (Raleigh, NC) for immunohistochemistry staining with Ki-67 assay. One slide represented one pig. Pictures were taken using a microscope (CX31, Olympus, Tokyo, Japan) and Infinity Analyze and Capture software (Lumenera Corporation, Ottawa, Canada).

Pictures were taken at magnification 40× to measure villus height (VH) and width (VW), crypt depth (CD), and villus height to crypt depth ratio (VH:CD). Lengths of 15 well-shaped villi and corresponding crypts were measured in each slide. Pictures of 15 well-shaped crypts were taken from each slide at magnification 100× and cropped to calculate the percentage of the Ki-67-positive cells to the number of epithelial cells in a crypt by operating the ImageJS (http://imagejs.org). The percentage of Ki-67-positive cells was used as an indicator of the enterocyte proliferation rate in the crypt (Chen et al., 2017; Duarte et al., 2019).

### Apparent ileal digestibility

Frozen ileal digesta samples were dried by a freeze dryer (24DX48, Virtis). Phase 2 diets and freeze dried ileal digesta were ground to fine powder form. The concentration of titanium dioxide in the diets and digesta was measured by following Myers et al. (2004). The DM was measured by following Passos et al. (2015). The gross energy (GE) was measured using a bomb calorimeter (Parr 6200, Parr Instrument Company; Moline, IL). Ether extract (EE) was analyzed by following AOAC (2006), method (920.39). Diets and ileal digesta samples were sent to Agricultural Experimental Station Chemical Laboratories, University of Missouri to analyze CP and AA without hydrolysis.

Apparent ileal digestibility of DM, GE, EE, CP, and AA was calculated as previously described by Chen et al. (2020) and Holanda and Kim (2020). The AID was calculated using the equation:

AID% = 100 × {1 – [(TiO_2_ in a diet/TiO_2_ in digesta) × (nutrient in digesta/nutrient in a diet)]

### Statistical analysis

All data except for fecal score were analyzed based on a randomized complete block design using Mixed procedure by SAS 9.3 (SAS Inc., Cary, NC). Initial BW and sex were considered as blocks. Dietary treatments were defined as fixed effects and blocks were random effects. The LSMEANS statement was used to calculate mean values for all treatments. Linear and quadratic effects of increasing CGCM supplementation were tested by orthogonal polynomial contrasts using the CONTRAST statement. When a quadratic effect was significant (*P* < 0.05), the procedure RSREG was used to predict the critical value and the stationary point. The linear and quadratic effects of daily intake (g/d) of CGCM were tested by REG procedures. The analysis of fecal score data was performed by using Kruskal–Wallis test with Dwass, Steel, Critchlow-Fligner method option for pairwise two-sided multiple comparisons following Guo et al. (2015). Statistical significance was *P* < 0.05 and 0.05 ≤ *P* < 0.10 were considered as a tendency.

## Results

### Lysis of CGCM

The process of homogenization using a French press increased (*P* < 0.05) the proportion of lysed *Corynebacterium glutamicum* cells from 4.3% in intact CGCM to 58% (Table 1).

### Growth performance and fecal score

**Figure 2.**
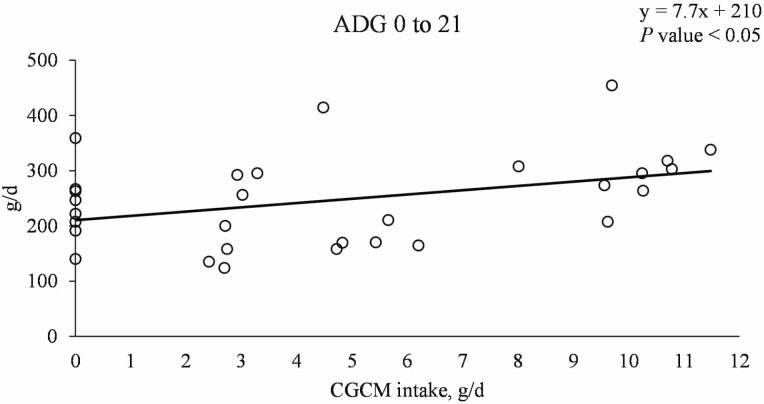
Average daily gain of nursery pigs with increasing daily intake of *Corynebacterium glutamicum* cell mass (CGCM). Linear model: *y* = 7.7*x* + 210, *P-*value < 0.05 (overall model), 0.039 (slope), and <0.0001 (intercept), *x* = daily CGCM intake (g/d), *y* = ADG (g/d).

Increasing CGCM supplementation did not affect the growth performance of nursery pigs (Table 3). However, when the growth performance data were analyzed based on CGCM intake (g/d), the ADG was linearly increased (*P* < 0.05; Figure 2).

**Table 3.** Growth performance of nursery pigs fed diets with increasing *Corynebacterium glutamicum* cell mass (CGCM) supplementation

	CGCM, %^1^					*P*-value	
Item	0	0.7	1.4	2.1	SEM	Linear	Quadratic
BW, kg							
Initial	7.1	7.1	7.2	7.2	0.6	0.569	0.964
Final, day 21	12.1	12.0	12.1	12.7	1.1	0.494	0.599
ADG, g	237	234	234	264	33	0.554	0.591
ADFI, g	433	433	456	471	36	0.378	0.820
G:F	0.55	0.53	0.50	0.55	0.04	0.910	0.309

^1^Four supplemental levels of CGCM (*n* = 32 total, *n* = 8 per supplemental level).

**Figure 3.**
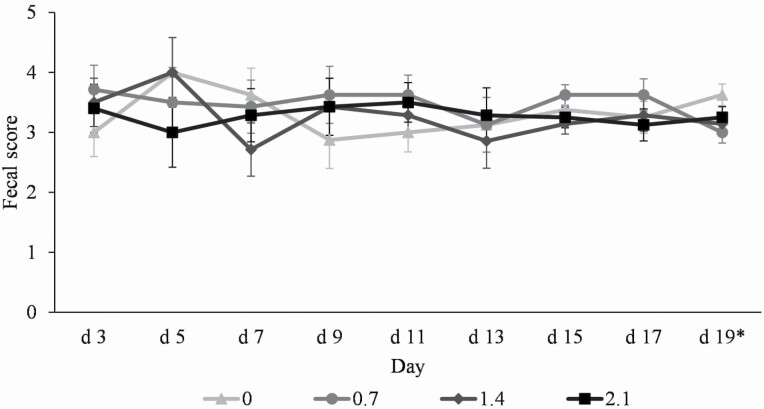
Fecal score of nursery pigs fed diets with increasing *Corynebacterium glutamicum* cell mass (CGCM) supplementation from 0% to 2.1% (0, 0.7, 1.4, and 2.1%). Fecal scores were: (1) very hard and dry stool, (2) firm stool, (3) normal stool, (4) loose stool, and (5) watery stool with no shape. *0 vs. 0.7: *P* < 0.05.

Pigs fed 0.7% CGCM had lower (*P* < 0.05) fecal score than pigs fed 0% CGCM on day 19 of the experiment, whereas no differences were observed among other treatments (Figure 3).

### Relative abundance and diversity of mucosa-associated microbiota

Increasing CGCM supplementation had quadratic effects (*P* < 0.05) on the alpha diversity of jejunal mucosa-associated microbiota estimated with Shannon index at family (maximum 2.8 at 1.0% CGCM), genus (maximum 2.7 at 1.0% CGCM), and species (maximum 3.8 at 1.0% CGCM) levels (Table 4). Increasing CGCM supplementation had quadratic effects (*P* < 0.05) on the alpha diversity of jejunal mucosa-associated microbiota estimated with Simpson index at family (maximum 0.8 at 1.1% CGCM), genus (maximum 0.7 at 1.1% CGCM), and species (maximum 0.9 at 1.1% CGCM) levels.

**Table 4.** Alpha-diversity of the mucosa-associated microbiota in the distal jejunum^1^ of nursery pigs fed diets with increasing *Corynebacterium glutamicum* cell mass (CGCM) supplementation

	CGCM, %^2^					*P*-value	
	0	0.7	1.4	2.1	SEM	Linear	Quadratic
Family							
Chao 1	44.6	46.9	44.0	49.1	6.1	0.702	0.819
Shannon	2.07	2.95	2.39	2.06	0.30	0.554	0.012
Simpson	0.57	0.77	0.69	0.61	0.07	0.891	0.007
Genus							
Chao 1	47.6	44.6	43.3	43.8	4.4	0.514	0.689
Shannon	2.09	2.88	2.35	1.95	0.32	0.349	0.016
Simpson	0.55	0.75	0.67	0.59	0.07	0.849	0.007
Species							
Chao 1	78.8	71.3	64.1	78.6	7.6	0.817	0.179
Shannon	3.09	3.84	3.58	3.04	0.32	0.663	0.017
Simpson	0.73	0.87	0.84	0.79	0.04	0.287	0.016

^1^Distal: 1.5 m before the ileocecal junction.

^2^Four supplemental levels of CGCM (*n* = 32 total, *n* = 8 per supplemental level).

**Figure 4.**
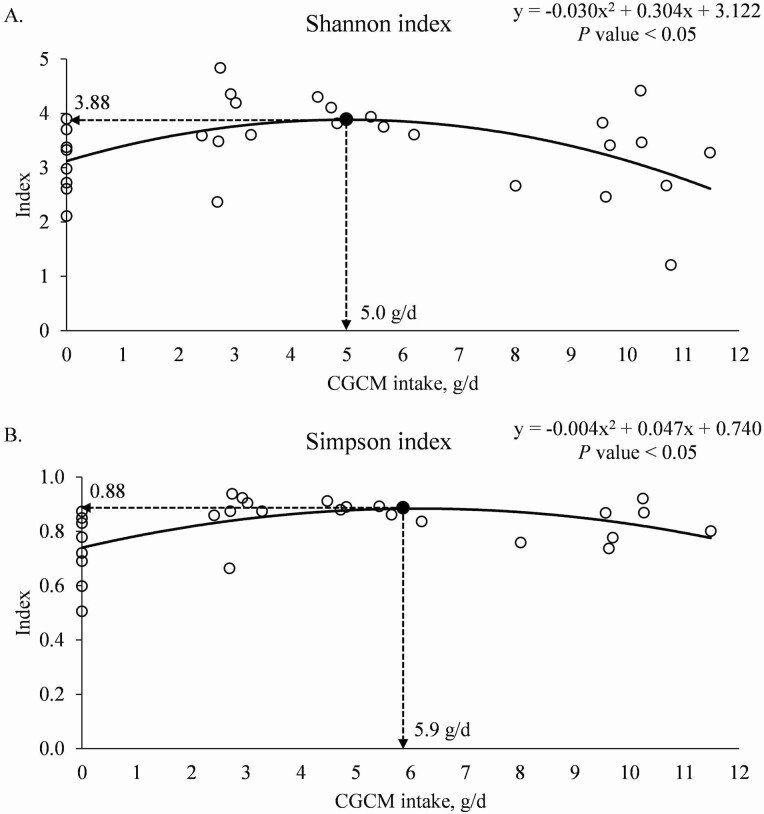
(a) Alpha diversity (Shannon index) of mucosa-associated microbiota at the species level in the distal jejunum of nursery pigs with increasing daily intake of *Corynebacterium glutamicum* cell mass (CGCM). Quadratic model: *y* = −0.030*x*^2^ + 0.304*x* + 3.122, *P-*value < 0.05 (overall model), 0.009 (*x*^2^), 0.017 (*x*), and <0.0001 (intercept), *x* = CGCM intake (g/d), *y* = Shannon index. (b). Alpha diversity (Simpson index) of mucosa-associated microbiota at the species level in the distal jejunum of nursery pigs with increasing daily intake of *Corynebacterium glutamicum* cell mass (CGCM). Quadratic model: *y* = −0.004*x*^2^ + 0.047*x* + 0.740, *P*-value < 0.05 (overall model), 0.007 (*x*^2^), 0.004 (*x*), and <0.0001 (intercept), *x* = CGCM intake, (g/d), *y* = Simpson index.

When the data were analyzed based on the daily CGCM intake (g/d), increasing the daily CGCM intake tended to have quadratic effects on the alpha diversity of jejunal mucosa-associated microbiota estimated with Simpson index (*P* = 0.094) at the family level with the maximum 0.75 at 5.4 g/d CGCM intake (quadratic model: *y* = −0.005*x*^2^ + 0.056*x* + 0.595; *R*² = 0.15). Increasing the daily CGCM intake tended to have the quadratic effects on the alpha diversity of jejunal mucosa-associated microbiota estimated with Shannon (*P* = 0.091) and Simpson (*P* = 0.079) indexes at the genus level with the maximum at 2.71 and 0.74, when the daily CGCM intakes were 4.7 (quadratic model: *y* = −0.025*x*^2^ + 0.234*x* + 2.159; *R*² = 0.16) and 5.4 g/d (quadratic model: *y* = −0.006*x*^2^ + 0.064*x* + 0.568; *R*² = 0.18), respectively. Increasing the daily CGCM intake had the quadratic effects (*P* < 0.05) on the alpha diversity of jejunal mucosa-associated microbiota estimated with Shannon and Simpson indexes at the species level with the maximum at 3.88 and 0.88, when the daily CGCM intake was 5.0 g/d (Figure 4a) and 5.9 g/d (Figure 4b), respectively.

**Figure 5.**
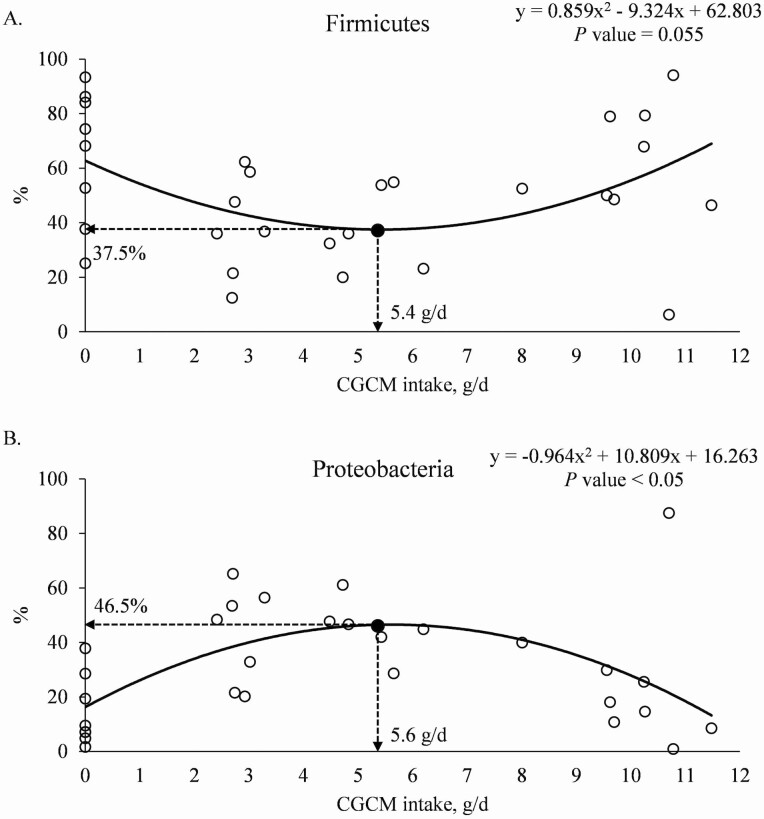
(a). Relative abundance of Firmicutes in the mucosa of distal jejunum of nursery pigs with increasing daily intake of *Corynebacterium glutamicum* cell mass (CGCM). Quadratic model: *y* = 0.859*x*^2^ – 9.324*x* + 62.803, *P-*value = 0.055 (overall model), 0.017 (*x*^2^), 0.0020 (*x*), and <0.0001 (intercept), *x* = CGCM intake (g/d), *y* = Firmicutes (%). (b). Relative abundance of Proteobacteria in the mucosa of distal jejunum of nursery pigs with increasing daily intake of *Corynebacterium glutamicum* cell mass (CGCM). Quadratic model: *y* = −0.964*x*^2^ + 10.809*x* + 16.263, *P-*value <0.05 (overall model), 0.002 (*x*^2^), 0.002 (*x*), and 0.016 (intercept), *x* = CGCM intake (g/d), *y* = Proteobacteria (%).

At the phylum level, increasing CGCM supplementation had quadratic effects (*P* < 0.05) on the minimum relative abundances of Firmicutes (minimum 37.8% at 1.1% CGCM) and Proteobacteria (maximum 44.0% at 1.1% CGCM) and tended to have a quadratic effect (*P* = 0.058) on the relative abundance of Actinobacteria (Table 5). When the data were analyzed based on the daily CGCM intake (g/d), increasing the daily CGCM intake tended to have a quadratic effect (*P* = 0.055) on the relative abundance of Firmicutes with minimum 37.5% at 5.4 g/d CGCM intake (Figure 5a). Increasing the daily CGCM intake had quadratic effect (*P* < 0.05) on the relative abundance of Proteobacteria with maximum abundance at 46.5%, when the daily CGCM intake was 5.6 g/d (Figure 5b).

**Table 5.** Relative abundance of mucosa-associated microbiota at the phylum level in the distal jejunum^1^ of nursery pigs fed diets with increasing *Corynebacterium glutamicum* cell mass (CGCM) supplementation

	CGCM, %^2^					*P*-value	
	0	0.7	1.4	2.1	SEM	Linear	Quadratic
Firmicutes	65.2	38.5	42.4	56.1	9.8	0.455	0.009
Bacteroidetes	17.3	9.2	13.8	16.1	6.9	0.962	0.301
Proteobacteria	14.5	43.2	39.1	25.3	6.7	0.353	0.005
Actinobacteria	2.5	4.6	4.2	2.3	1.1	0.820	0.058
Spirochaetes	0.6	6.8	0.3	0.1	2.5	0.430	0.201
Others^3^	0.2	0.3	0.7	0.2	0.3	0.750	0.268
F/B ratio^4^	13.7	14.0	7.3	25.4	7.8	0.365	0.207

^1^Distal jejunum: 1.5 m before the ileocecal junction.

^2^Four supplemental levels of CGCM (*n* = 32 total, *n* = 8 per supplemental level).

^3^The OTU with the relative abundance < 1.0% within each level was combined in each phlyum.

^4^F/B ratio, firmicutes to bacteroidetes ratio.

At the family level, increasing CGCM supplementation had quadratic effects (*P* < 0.05) on the relative abundance of *Lactobacillaceae* (minimum 26.4% at 1.2% CGCM), *Helicobacteraceae* (maximum 29.3% at 1.2% CGCM), and *Campylobacteraceae* (maximum 9.0% at 1.0% CGCM) (Table 6). Increasing CGCM supplementation tended to have a quadratic effect (*P* = 0.060) on the relative abundance of *Propionibacteriaceae*, whereas linearly reduced (*P* < 0.05) the relative abundance of *Bifidobacteriaceae*. When the data were analyzed based on the daily CGCM intake (g/d), increasing the daily CGCM intake tended to have quadratic effects on the relative abundance of *Helicobacteraceae* (*P* = 0.053) and *Campylobacteraceae* (*P* = 0.098) with minimum abundance at 31.3% and 9.0% when the daily CGCM intake was 5.8 g/d (quadratic model: *y* = 0.692*x*^2^ + 8.027*x* + 8.036; *R*² = 0.20) and 5.1 g/d (quadratic model: *y* = −0.284*x*^2^ + 2.867*x* + 1.735; *R*² = 0.16), respectively. Increasing the daily CGCM intake tended to linearly reduce (*P* = 0.067) the relative abundance of *Bifidobacteriaceae* (linear model: *y* = −0.074*x* + 1.514; *R*² = 0.11).

**Table 6.** The relative abundance of mucosa-associated microbiota at the family level in the distal jejunum^1^ of nursery pigs fed diets with increasing *Corynebacterium glutamicum* cell mass (CGCM) supplementation

	CGCM, %^2^					*P*-value	
	0	0.7	1.4	2.1	SEM	Linear	Quadratic
*Lactobacillaceae*	51.2	23.3	34.6	37.4	11.2	0.364	0.048
*Helicobacteraceae*	7.0	25.7	28.3	17.7	6.7	0.263	0.042
*Prevotellaceae*	17.1	8.7	13.2	15.4	6.7	0.988	0.272
*Veillonellaceae*	9.8	6.1	6.6	7.3	3.0	0.546	0.413
*Campylobacteraceae*	0.9	10.5	5.6	0.7	2.9	0.667	0.022
*Clostridiaceae*	0.9	3.1	0.0	3.0	1.9	0.638	0.772
*Succinivibrionaceae*	2.6	0.6	1.7	1.0	1.4	0.455	0.563
*Peptostreptococcaceae*	0.4	2.0	0.0	3.3	2.2	0.397	0.609
*Pasteurellaceae*	1.3	0.3	1.0	3.0	1.8	0.406	0.355
*Bifidobacteriaceae*	1.5	1.5	1.1	0.6	0.4	0.029	0.419
*Corynebacteriaceae*	0.3	1.4	1.8	1.1	0.6	0.253	0.149
*Streptococcaceae*	1.2	1.2	0.3	0.9	0.5	0.417	0.528
*Propionibacteriaceae*	0.4	1.0	1.0	0.4	0.3	0.870	0.060
*Pseudomonadaceae*	0.2	1.1	0.2	0.5	0.4	0.994	0.410
Others^3^	5.2	13.7	6.1	7.7	2.2	0.995	0.145

^1^Distal jejunum: 1.5 m before the ileocecal junction.

^2^Four supplemental levels of CGCM (*n* = 32 total, *n* = 8 per supplemental level).

^3^The OTU with the relative abundance < 1.0% within each level was combined in each family.

At the genus level, increasing CGCM supplementation tended to have quadratic effects on the relative abundance of *Lactobacillus* (*P* = 0.056) and *Propionibacterium* (*P* = 0.073) and had quadratic effects (*P* < 0.05) on the relative abundance of *Helicobacter* (31.9%) and *Campylobacter* (9.4%) at 1.2% and 1.0% CGCM, respectively (Table 7). Increasing CGCM supplementation tended to linearly reduce (*P* = 0.068) the relative abundance of *Megasphaera* and linearly reduced (*P* < 0.05) relative abundance of *Bifidobacterium*. When the data were analyzed based on the daily CGCM intake (g/d), increasing the daily CGCM intake had a quadratic effect (*P* < 0.05) on the relative abundance of *Helicobacter* with 33.9% at 5.7 g/d CGCM intake (quadratic model: *y* = −0.737*x*^2^ + 8.368*x* + 10.102; *R*² = 0.20). Increasing the daily CGCM intake tended to linearly reduce (*P* = 0.064) the relative abundance of *Bifidobacterium* (linear model: *y* = −0.086*x* + 1.625; *R*² = 0.12).

**Table 7.** The relative abundance of mucosa-associated microbiota at the genus level in the distal jejunum^1^ of nursery pigs fed diets with increasing *Corynebacterium glutamicum* cell mass (CGCM) supplementation

	CGCM, %2					*P*-value	
	0	0.7	1.4	2.1	SEM	Linear	Quadratic
*Lactobacillus*	54.2	26.0	37.1	38.4	11.3	0.271	0.056
*Helicobacter*	8.4	30.4	28.8	19.1	6.9	0.331	0.033
*Prevotella*	13.9	8.1	11.4	16.1	7.7	0.649	0.297
*Campylobacter*	0.8	10.4	6.7	0.8	3.3	0.806	0.031
*Mitsuokella*	4.3	1.4	2.3	2.1	1.1	0.270	0.250
*Clostridium*	0.8	3.7	0.0	4.3	3.0	0.517	0.730
*Selenomonas*	1.2	1.7	1.1	4.1	1.9	0.320	0.503
*Megasphaera*	3.7	1.4	1.1	0.6	1.2	0.068	0.435
*Succinivibrio*	2.6	0.6	1.6	0.9	1.4	0.416	0.581
*Actinobacillus*	1.0	0.3	1.0	3.3	1.9	0.337	0.387
*Corynebacterium*	0.3	1.7	2.1	1.3	0.8	0.309	0.142
*Bifidobacterium*	1.5	1.6	1.1	0.6	0.4	0.029	0.410
*Propionibacterium*	0.5	1.3	1.1	0.5	0.4	0.866	0.073
*Pseudomonas*	0.2	1.3	0.2	0.5	0.4	0.938	0.355
Others^3^	6.7	10.2	6.4	7.5	2.3	0.880	0.532

^1^Distal jejunum: 1.5 m before the ileocecal junction.

^2^Four supplemental levels of CGCM (*n* = 32 total, *n* = 8 per supplemental level).

^3^The OTU with the relative abundance < 1.0% within each level was combined in each genus.

**Figure 6.**
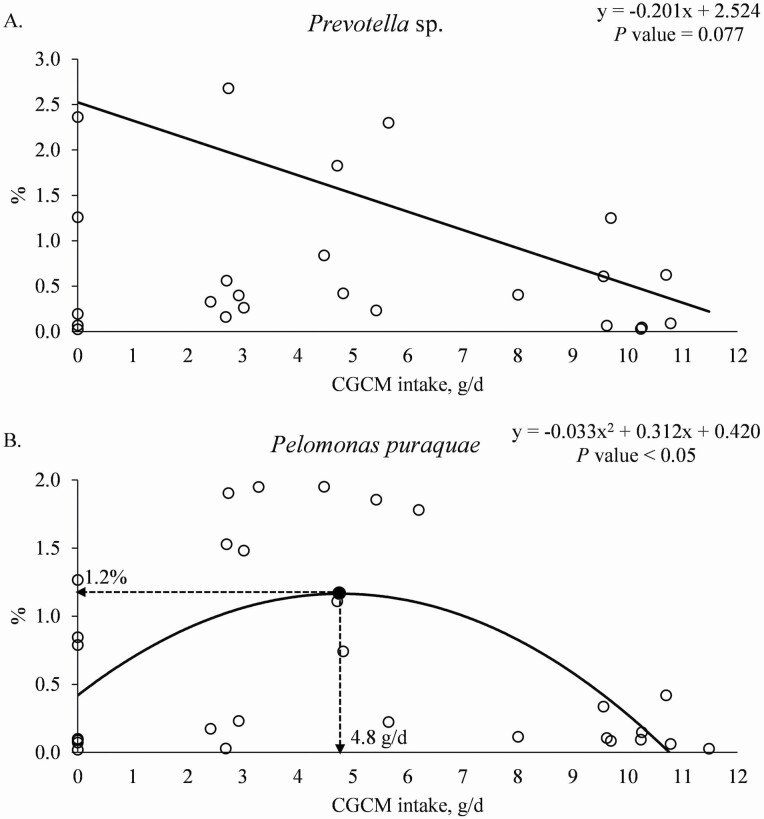
(a). Relative abundance of *Prevotella* sp. in the mucosa of distal jejunum of nursery pigs with increasing daily intake of *Corynebacterium glutamicum* cell mass (CGCM). Linear model: *y* = −0.201*x* + 2.524, *P-*value = 0.077 (overall model), 0.077 (slope), and 0.001 (intercept), *x* = CGCM intake (g/d), *y* = *Prevotella* sp. (%). (b). Relative abundance of *Pelomonas puraquae* in the mucosa of distal jejunum of nursery pigs with increasing daily intake of *Corynebacterium glutamicum* cell mass (CGCM). Quadratic model: *y* = −0.033*x*^2^ + 0.312*x* + 0.420, *P-*value < 0.05 (overall model), 0.001 (*x*^2^), 0.004 (*x*), and 0.047 (intercept), *x* = CGCM intake (g/d), *y* = *Pelomonas puraquae* (%).

At the species level, increasing CGCM supplementation linearly reduced (*P* = 0.023) the relative abundance of *Prevotella* sp. (Table 8). Increasing CGCM supplementation tended to linearly reduce (*P* = 0.096) and tended to have a quadratic effect (*P* = 0.090) on the relative abundance of *Lactobacillus kitasatonis* and tended to linearly reduce (*P* = 0.053) the relative abundance of *Corynebacterium glutamicum*. Increasing CGCM supplementation had a quadratic effect (*P* < 0.05) on the relative abundance of *Pelomonas puraquae* (maximum 1.1% at 1.0% CGCM). When the data were analyzed based on the daily CGCM intake (g/d), increasing the daily CGCM intake tended to linearly reduce (*P* = 0.077) the relative abundance of *Prevotella* sp. (Figure 6a). Increasing the daily CGCM intake had a quadratic effect (*P* < 0.05) on the relative abundances of *Pelomonas puraquae* with minimum abundance at 1.2%, when the daily CGCM intake was 4.8 g/d (Figure 6b).

**Table 8.** The relative abundance of mucosa-associated microbiota at the species level in the distal jejunum^1^ of nursery pigs fed diets with increasing *Corynebacterium glutamicum* cell mass (CGCM) supplementation

	CGCM, %^2^					*P*-value	
	0	0.7	1.4	2.1	SEM	Linear	Quadratic
*Lactobacillus kitasatonis*	33.1	10.9	16.8	21.3	10.4	0.096	0.090
*Prevotella copri*	11.7	10.2	17.8	17.1	8.8	0.390	0.440
*Helicobacter rappini*	4.6	13.1	10.1	7.6	2.8	0.201	0.113
*Lactobacillus mucosae*	9.7	8.1	7.8	5.0	3.1	0.512	0.794
*Helicobacter mastomyrinus*	3.6	8.9	7.3	7.4	3.7	0.494	0.439
*Lactobacillus delbrueckii*	4.1	2.7	2.5	6.3	2.3	0.567	0.815
*Campylobacter upsaliensis*	0.2	5.2	6.2	0.5	2.5	0.117	0.532
*Mitsuokella jalaludinii*	4.8	1.1	2.6	1.7	1.3	0.226	0.107
*Campylobacter coli*	0.6	7.1	0.2	0.2	3.4	0.943	0.124
*Prevotella stercorea*	2.0	1.5	2.1	2.2	1.3	0.931	0.630
*Dialister succinatiphilus*	1.7	1.2	3.0	0.3	0.9	0.350	0.358
*Prevotella* sp.	3.4	0.7	0.8	1.1	0.9	0.023	0.125
*Propionibacterium acnes*	0.8	2.1	2.2	0.9	0.7	0.141	0.408
*Selenomonas bovis*	0.8	1.5	0.9	2.6	1.4	0.959	0.696
*Corynebacterium glutamicum*	0.2	1.8	2.3	1.2	0.7	0.053	0.533
*Lactobacillus* sp.	0.5	0.9	0.9	2.7	1.3	0.816	0.927
*Succinivibrio dextrinosolvens*	2.3	0.3	1.2	0.9	1.5	0.502	0.327
*Selenomonas lipolytica*	0.8	0.9	0.8	1.9	0.9	0.968	0.922
*Pelomonas puraquae*	0.4	1.2	0.9	0.2	0.3	0.119	0.040
*Helicobacter canadensis*	0.4	0.0	0.0	0.0	0.2	0.242	0.412
Others^3^	14.2	20.6	14.8	18.9	5.1	0.933	0.325

^1^Distal jejunum: 1.5 m before the ileocecal junction.

^2^Four supplemental levels of CGCM (*n* = 32 total, *n* = 8 per supplemental level).

^3^The OTU with the relative abundance < 1.0% within each level was combined in each species.

### Immune status and oxidative stress

**Figure 7.**
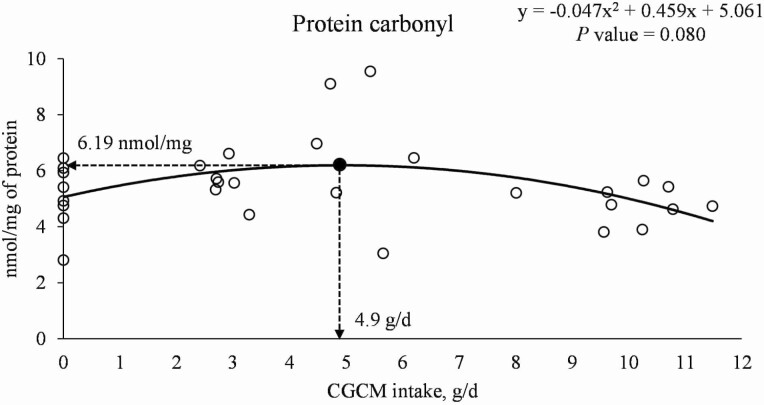
The change of protein carbonyl (PC) in the mucosa of proximal jejunum of nursery pigs with increasing daily intake of *Corynebacterium glutamicum* cell mass (CGCM). Quadratic model: *y* = −0.047*x*^2^ + 0.459*x* + 5.061, *P-*value = 0.080 (overall model), 0.031 (*x*^2^), 0.053 (*x*), and <0.0001 (intercept), *x* = CGCM intake (g/d), *y* = PC (nmol/mg of protein).

In the proximal jejunal mucosa, increasing CGCM supplementation did not affect the concentration of TNFα and IL-8 in nursery pigs (Table 9). Increasing CGCM supplementation tended to have a quadratic effect (*P* = 0.051) on the concentration of IgA and had quadratic effects (*P* < 0.05) on the concentrations of IgG (maximum 4.94 µg/mg of protein at 1.0% CGCM) and PC (maximum 6.12 nmol/mg of protein at 1.1% CGCM). Whereas increasing CGCM supplementation linearly reduced (*P* < 0.05) the concentration of MDA. When the data were analyzed based on daily CGCM intake (g/d), increasing the daily intake of CGCM tended to have a quadratic effect (*P* = 0.080) on the concentration of PC with maximum 6.19 nmol/mg of protein at 4.9 g/d CGCM intake (Figure 7).

**Table 9.** Immune and oxidative stress markers in the jejunal mucosa of nursery pigs fed diets with increasing *Corynebacterium glutamicum* cell mass (CGCM) supplementation

	CGCM, %^1^					*P*-value	
Item	0	0.7	1.4	2.1	SEM	Linear	Quadratic
Proximal jejunal mucosa^2^, amount/mg of protein							
TNFα^3^, pg	0.83	0.62	0.74	0.75	0.12	0.786	0.284
IL-8^4^, ng	0.49	0.56	0.54	0.47	0.07	0.709	0.241
IgA^5^, µg	3.06	4.08	5.39	3.20	0.78	0.621	0.051
IgG^6^, µg	2.26	2.91	3.61	1.99	0.47	0.958	0.025
MDA^7^, µmol/g	0.64	0.51	0.51	0.47	0.07	0.028	0.382
PC^8^, nmol	5.09	5.80	6.22	5.07	0.64	0.860	0.047
Distal jejunal mucosa^9^, amount/mg of protein							
TNFα, pg	2.36	2.55	2.29	1.82	0.33	0.219	0.327
IL-8, ng	0.80	0.77	0.98	0.56	0.12	0.297	0.098
IgA, µg	3.56	3.62	4.00	2.59	0.73	0.360	0.246
IgG, µg	3.32	3.17	4.07	2.63	0.66	0.687	0.326
MDA, µmol/g	0.69	0.70	0.67	0.55	0.10	0.292	0.471
PC, nmol	4.93	5.95	6.56	5.06	0.83	0.717	0.051

^1^Four supplemental levels of CGCM (*n* = 32 total, *n* = 8 per supplemental level).

^2^Proximal jejunum: 1.5 m after the pyloric duodenal junction.

^3^ TNFα, tumor necrosis factor alpha.

^4^IL-8, interleukin 8.

^5^IgA, immunoglobulin A.

^6^IgG, immunoglobulin G.

^7^MDA, malondialdehyde.

^8^PC, protein carbonyl.

^9^Distal: 1.5 m before the ileocecal junction.

In the distal jejunal mucosa, increasing CGCM supplementation did not affect the concentration of TNFα, IgA, IgG, and MDA in nursery pigs (Table 9). Increasing CGCM supplementation tended to have quadratic effects on the concentration of IL-8 (*P* = 0.098) and PC (*P* = 0.051).

### Intestinal morphology and crypt cell proliferation

Increasing CGCM supplementation did not affect VH, VW, and CD in proximal and distal jejunum (Table 10). Whereas increasing CGCM supplementation tended to linearly reduce (*P* = 0.090) VH:CD in the proximal jejunal mucosa and had a quadratic effect (*P* < 0.05) on the maximum intestinal enterocyte proliferation rate (13.3%) at 1.0% CGCM in the distal jejunal mucosa.

**Table 10.** Proximal and distal intestinal morphology and enterocyte proliferation of nursery pigs fed diets with increasing *Corynebacterium glutamicum* cell mass (CGCM) supplementation

	CGCM, %^1^					*P*-value	
Item	0	0.7	1.4	2.1	SEM	Linear	Quadratic
Proximal^2^ jejunal tissue							
Villus height, μm	509	447	481	444	27	0.190	0.649
Villus width, μm	121	118	117	116	6	0.451	0.890
Crypt depth, μm	257	239	277	264	46	0.439	0.878
VH:CD^3^	2.05	1.96	1.86	1.74	0.33	0.090	0.905
Ki-67^+^^4^, %	10.1	10.9	12.7	10.6	2.1	0.581	0.292
Distal^5^ jejunal tissue							
Villus height, μm	307	327	291	386	31	0.165	0.233
Villus width, μm	97	97	96	106	5	0.189	0.288
Crypt depth, μm	262	277	281	288	15	0.257	0.796
VH:CD	1.17	1.23	1.05	1.39	0.11	0.357	0.214
Ki-67^+^, %	10.3	13.4	12.6	10.3	1.5	0.833	0.004

^1^Four supplemental levels of CGCM (*n* = 32 total, *n* = 8 per supplemental level).

^2^Proximal jejunum: 1.5 m after the pyloric duodenal junction.

^3^VH:CD, villus height to crypt depth ratio.

^4^Ki-67^+^, enterocyte proliferation rate in crypt.

^5^Distal jejunum: 1.5 m before the ileocecal junction.

### Apparent ileal digestibility

Increasing CGCM supplementation did not affect the AID of DM, GE, EE, CP, and AA in nursery pig diets (Table 11).

**Table 11.** Apparent ileal digestibility of nutrients in diets (dry matter basis) with increasing *Corynebacterium glutamicum* cell mass (CGCM) supplementation fed to nursery pigs

	CGCM, %^1^					*P* value	
Item, %^2^	0	0.7	1.4	2.1	SEM	Linear	Quadratic
DM	48.8	36.6	55.8	43.3	5.3	0.908	0.978
GE	57.5	46.6	65.2	54.6	5.1	0.629	0.972
EE	66.9	44.3	66.2	61.0	5.6	0.849	0.139
CP	74.6	68.4	71.7	71.6	3.1	0.698	0.372
Indispensable AA							
Arg	83.4	78.8	81.7	80.4	2.2	0.542	0.484
His	89.2	87.2	88.9	88.6	1.3	0.977	0.545
Ile	82.5	78.4	80.5	78.3	2.2	0.293	0.681
Leu	64.3	62.6	68.5	64.2	3.6	0.742	0.747
Lys	77.7	75.7	75.2	74.4	2.8	0.386	0.836
Met	93.5	92.7	92.4	92.7	1.2	0.518	0.558
Phe	81.2	77.0	79.8	78.0	2.5	0.521	0.639
Thr	79.6	77.2	77.1	76.6	2.7	0.370	0.681
Trp	95.9	95.0	95.4	95.9	0.6	0.895	0.291
Val	76.8	71.4	77.8	73.8	2.3	0.810	0.770
Dispensable AA							
Ala	73.4	66.3	65.8	65.6	4.4	0.161	0.363
Asp	59.4	50.8	48.5	52.7	6.1	0.395	0.296
Cys	87.8	84.5	86.0	87.9	1.7	0.798	0.147
Glu	35.9	39.7	35.2	37.4	8.6	0.999	0.917
Gly	66.6	63.2	64.4	62.6	4.1	0.566	0.865
Pro	71.5	64.4	68.9	68.5	4.0	0.783	0.399
Ser	81.5	76.8	80.2	77.2	2.4	0.398	0.745
Tyr	87.2	83.5	86.3	85.8	1.7	0.861	0.396

^1^Four supplemental levels of CGCM (*n* = 32 total, *n* = 8 per supplemental level).

^2^DM, dry matter; GE, gross energy; EE, ether extract; AA, amino acids.

## Discussion

This study demonstrated that CGCM can be supplemented up to 2.1%, replacing 1.5% blood plasma, in diets without affecting the growth performance and intestinal health of nursery pigs. Additionally, increasing daily CGCM intake increased the ADG of pigs. The results observed in this study are in accordance with previous studies showing that SCP can be used as alternative protein supplements to replace plant or animal protein supplements improving growth performance in broilers (Schøyen et al., 2007; Chand and Khan, 2014) and pigs (Hu et al., 2014; Sampath et al., 2021). Furthermore, blood plasma has high protein and energy digestibility in diets (Chae et al., 1999; Jeong et al., 2016); however, replacing blood plasma by increasing CGCM supplementation did not affect nutrient digestibility of diets, indicating that supplementing 2.1% CGCM can replace 1.5% blood plasma without compromising the nutrient digestibility in diets fed to nursery pigs.

Previous studies have demonstrated that diet composition greatly affected the intestinal microbiota (Etheridge et al., 1984; Upadrasta et al., 2013; Niu et al., 2015). Interestingly, in this study, the increasing CGCM supplementation, replacing blood plasma, showed a quadratic effect on the relative abundance of Firmicutes and Proteobacteria, two of the dominant microbiota phyla in the jejunal mucosa of pigs (Adhikari et al., 2019; Duarte et al., 2021). The supplementation of CGCM up to 1.1% or 5.6 g/d increased the relative abundance of Proteobacteria and reduced the relative abundance of Firmicutes. Conversely, supplementing 2.1% CGCM in diets reduced the relative abundance of Proteobacteria and increased the relative abundance of Firmicutes. The relative abundance of *Helicobacteraceae,* which belongs to Proteobacteria, is commonly associated to unhealthy pigs (Zhang et al., 2017; Vigors et al., 2019; Duarte et al., 2020), whereas increased relative abundance of *Lactobacillaceae* in Firmicutes is predominant in healthy pigs (Zhang et al., 2018; Adhikari et al., 2019).

Blood plasma not only is highly digestible but also provides around 20% of IgG that plays an essential role in the immune response by affecting the host immune system and intestinal microbiota (Tran et al., 2014, 2018). Igs from blood plasma bind to potential antigens in the small intestinal lumen and limit the adherence and colonization of potential pathogens, resulting in lower activation of immune response (Touchette et al., 2002). Furthermore, previous studies showed that blood plasma supplemented in diets for pigs (Tran et al., 2018) and broilers (Campbell et al., 2019) increased the relative abundance of *Lactobacillaceae*, which can increase the production of anti-inflammatory cytokines and, consequently, reduce proinflammatory cytokines (Macia et al., 2012). The cell wall of Gram-positive bacteria, including *Corynebacterium glutamicum*, contains TA and Slp that can affect the intestinal microbiota by immunomodulatory effects (Katayama et al., 2011; Poulsen et al., 2018) and by competitive exclusion (Johnson-henry et al., 2007; Oelschlaeger, 2010). Previous studies reported that the Slp isolated from cell wall of *Lactobacillus* attaches to receptors on the intestinal epithelial cells and, consequently, reduces the availability of receptors for the adherence of potential pathogens (Scheuring et al., 2002; Johnson-henry et al., 2007). In this study, based on the orthogonal polynomial model used in the statistical analysis, supplementing CGCM at 0% to 1.1%, replacing 0% to 0.7% blood plasma, reduced available Ig from blood plasma, whereas TA and Slp were not sufficiently provided from CGCM yet limiting functional benefits of CGCM on the intestinal microbiota. The insufficient availability of Ig, TA, and Slp when supplementing 1.1% or 5.6 g/d CGCM resulted in an increased relative abundance of Proteobacteria mainly by increasing the abundance of *Helicobacteraceae.* However, supplementing 2.1% CGCM, replacing 1.5% blood plasma, in diets may have provided sufficient TA and Slp for their functional roles reducing the relative abundance of Proteobacteria and increasing Firmicutes in the jejunal mucosa.

The alpha diversity of intestinal microbiota can also be affected by the diet composition (Power et al., 2014; Baker et al., 2021). The carbohydrates, including PGN and TA, in the cell wall of Gram-positive bacteria can be used as sources of energy by intestinal microbiota and affect the diversity of the gut microbiota in humans and pigs (Kaoutari et al., 2013; Kogut and Arsenault, 2016). High diversity is usually correlated with healthy status (Bhandari et al., 2008; Fouhse et al., 2016; Kim and Duarte, 2021). However, in this study, the highest diversity was associated with the highest abundance of potential harmful bacteria and highest immune response. According to Tran et al. (2018), it is unclear to decide whether increased or reduced alpha diversity of fecal microbiota is beneficial to intestinal health, therefore further assessment of microbial composition at different taxonomic levels is necessary.

The modulation of intestinal microbiota is generally associated with changes in the immune response (Duarte and Kim, 2021). The effects of increasing CGCM supplementation on the relative abundance of the microbiota observed in this study can explain the results on the immune and the oxidative stress status. The increased relative abundance of Proteobacteria, including *Helicobacteraceae* and *Campylobacteraceae*, can affect the immune system and oxidative stress status in the jejunum of nursery pigs (McOrist et al., 1992; Aiba et al., 1998). In response to Gram-negative bacteria, including the Proteobacteria, the intestinal immune cells produce IgG to protect the host against systemic infection (Zeng et al., 2016). Conversely, previous studies showed that greater relative abundance of *Lactobacillaceae* can be associated with the synthesis of anti-inflammatory cytokines (Tran et al., 2018; Campbell et al., 2019).

Previous study showed that pigs fed blood plasma in diets improved intestinal health by preventing from the production of proinflammatory cytokines, such as TNFα, IL-8, and interferon-γ, resulting in apoptosis of infected cells and the production of additional proinflammatory cytokines and chemokines (Tran et al., 2014). Therefore, blood plasma supplementation would be efficiently utilized for growth by enhancing intestinal health and nutrient utilization (Pérez-Bosque et al., 2004; Pierce et al., 2005). The cell wall of Gram-positive bacteria, including *Corynebacterium glutamicum*, contains CWGs that have immunomodulatory functions, reducing excessive immune reactions (Weidenmaier and Peschel, 2008; Yasuda et al., 2008). The CWGs from bacterial cell wall bind to the immune cells, including dendritic cells and macrophages, and then activate innate and adaptive immune response (Weidenmaier and Peschel, 2008). According to Ha et al. (2006), PGN derived from bacterial wall activates the production of IgA by receptors on the innate intestinal epithelium in mice. It is well known that Ig can reduce inflammatory status in nursery pigs (Bosi et al., 2004). In the current study, supplemental CGCM at 1.0% reducing blood plasma supplementation by 0.7%, in diets caused increased immune response, whereas supplemental CGCM at 2.1%, reducing blood plasma supplementation by 1.5%, in diets did not affect intestinal health compared to pigs fed diets without CGCM. This result may indicate that supplemental CGCM at 2.1% in diets may overcome the increased immune response caused by the reduction of supplemental blood plasma at 1.5% due to the modulation of the jejunal mucosa-associated microbiota.

The activation of the immune system can increase the production of reactive oxygen species leading to an increase in oxidative stress (Gilljam et al., 2020). Malondialdehyde and PC are considered biomarkers of oxidative stress (Shacter, 2000). In the current study, pigs fed diets with increasing CGCM supplementation had reduced the concentration of MDA, indicating less lipid peroxidation in the proximal jejunal mucosa. Supplementing 1.1% or 4.9 g/d CGCM had the highest concentration of PC in proximal jejunal mucosa which can be associated with the greater immune response caused by the increased abundance of Proteobacteria at 1.1% CGCM.

The oxidative stress can cause damage in the enterocytes by oxidizing lipids, proteins, and DNA resulting in cellular apoptosis, consequently, affecting the intestinal morphology (Sido et al., 2017; Duarte et al., 2019). Increasing cell death is associated to a greater crypt cell proliferation (Pluske et al., 1997). In this study, the maximum enterocyte proliferation was observed with the supplementation 1.0% CGCM. Increased intestinal enterocyte proliferation rate results in reducing the nutrient utilization due to more immature cells with lower digestive enzyme activities (Håkenåsen et al., 2020). Additionally, intestinal integrity may affect the digestion and absorption capability of monogastric animals (Montagne et al., 2003; Holanda and Kim, 2020); however, in this study, increasing CGCM supplementation did not affect the AID of nutrients of diets fed to pigs.

In conclusion, based on the polynomial model, supplementing CGCM at 1.0% to 1.2%, reducing the blood plasma supplementation by 0.7% to 0.9%, respectively, increased potential pathogenic microbiota resulting in increased immune response, enterocyte proliferation, and PC concentration. However, supplementing CGCM at 2.1%, reducing blood plasma supplementation by 1.5%, improved growth performance, and reduced MDA concentration without affecting nutrient digestibility, intestinal morphology, and microbiota in the jejunal mucosa. Collectively, supplementing 1.0% to 1.2% CGCM suppressed the benefits from blood plasma, whereas supplementing 2.1% CGCM showed functional benefits of CGCM with similar effects from blood plasma supplementation.

## Abbreviations

AA: amino acid(s)
ADFI: average daily feed intake
ADG: average daily gain
AID: apparent ileal digestibility
CD: crypt depth
DM: dry matter
EE: ether extract
GE: gross energy
G:F: gain-to-feed ratio
IBW: initial body weight
MDA: malondialdehyde
ME: metabolizable energy
PGN: peptidoglycan
SCP: single cell protein
SID: standard ileal digestibility
Slp: surface-layer protein
STTD: standardized total tract digestible
TA: teichoic acid
